# The characteristics of spatial-temporal distribution and cluster of tuberculosis in Yunnan Province, China, 2005–2018

**DOI:** 10.1186/s12889-019-7993-5

**Published:** 2019-12-21

**Authors:** Jinou Chen, Yubing Qiu, Rui Yang, Ling Li, Jinglong Hou, Kunyun Lu, Lin Xu

**Affiliations:** Division of tuberculosis control and prevention, Yunnan Center for Disease Control and Prevention, Kunming, Yunnan China

**Keywords:** Tuberculosis, Spatial-temporal cluster, Scan statistics, Yunnan, Greater Mekong subregion

## Abstract

**Background:**

Tuberculosis (TB) makes a big challenge to public health, especially in high TB burden counties of China and Greater Mekong Subregion (GMS). The aim of this study was to identify the spatial-temporal dynamic process and high-risk region of notified pulmonary tuberculosis (PTB), sputum smear-positive tuberculosis (SSP-TB) and sputum smear-negative tuberculosis (SSN-TB) cases in Yunnan, the south-western of China between years of 2005 to 2018. Meanwhile, to evaluate the similarity of prevalence pattern for TB among GMS.

**Methods:**

Data for notified PTB were extracted from the China Information System for Disease Control and Prevention (CISDCP) correspond to population information in 129 counties of Yunnan between 2005 to 2018. Seasonally adjusted time series defined the trend cycle and seasonality of PTB prevalence. Kulldorff’s space-time scan statistics was applied to identify temporal, spatial and spatial-temporal PTB prevalence clusters at county-level of Yunnan. Pearson correlation coefficient and hierarchical clustering were applied to define the similarity of TB prevalence among borders with GMS.

**Result:**

There were a total of 381,855 notified PTB cases in Yunnan, and the average prevalence was 59.1 per 100,000 population between 2005 to 2018. A declined long-term trend with seasonality of a peak in spring and a trough in winter for PTB was observed. Spatial-temporal scan statistics detected the significant clusters of PTB prevalence, the most likely cluster concentrated in the northeastern angle of Yunnan between 2011 to 2015 (*RR* = 2.6, *P* < 0.01), though the most recent cluster for PTB and spatial cluster for SSP-TB was in borders with GMS. There were six potential TB prevalence patterns among GMS.

**Conclusion:**

This study detected aggregated time interval and regions for PTB, SSP-TB, and SSN-TB at county-level of Yunnan province. Similarity prevalence pattern was found in borders and GMS. The localized prevention strategy should focus on cross-boundary transmission and SSN-TB control.

## Background

Tuberculosis (TB) is a communicable disease caused by the agent of *Mycobacterium tuberculosis,* which mainly invade lung tissues and lead to airborne infectious disease of pulmonary tuberculosis (PTB).

TB made a big challenge to public health especially in high disease burden counties [1, 2]. Globally, high TB burden counties were primarily concentrated in Asia and Africa. World Health Organization (WHO) estimated there were 10.0 million TB cases worldwide, TB incidence was 889,000 and a rate of 63 per 100,000 population for China in 2017 [3]. Especially, Five out of six Greater Mekong Subregion (GMS) countries in southeast Asia were defined as high epidemiological TB burden, thus, Myanmar, Lao People’s Democratic Republic (Laos) and Vietnam shared not only national boundaries but also TB burden with China.

Although nation-wide longitudinal TB prevalence surveys showed that prevalence of PTB and the most infectious sputum smear-positive tuberculosis (SSP-TB) substantially decreased through two decades efforts and intensive directly observed treatment, short-course strategy (DOTS) programme [4], China still has the second-largest burden of TB cases in the world. Previous statistical modeling analysis by applying time series method reported that the trend for notified TB cases decreased in China between 2005 to 2012 [5], but long-term trend various in different provinces in the nation [6, 7]. Yunnan Province followed the National Tuberculosis Control Program in China (China NTP), yet implemented non-routine strategy of active case finding to detect TB cases in hard-to-reach population among few communities [8]. This public health action may have an impact on local TB epidemic status and made it a challenge to describe and understand the trend of TB prevalence.

As an airborne disease, PTB epidemics influenced the transmission in geographical neighborhoods, disease hotspots were defined as high-risk clusters. Studies reported hotspots regions or high-risk clusters of TB in China by using spatial-temporal scan analysis [9–14], spatial-temporal distribution characteristics were illustrated at the national, provincial, prefectural, county-level or individual level in diverse time frames, all of which have shown the geographical and temporal heterogeneity of TB epidemic.

The geospatial difference of TB prevalence distribution was observed for Yunnan in 2018, the highest notification rate of Lanping county was 28 times higher than the lowest rate of Tonghai county [15]. Yunnan province was in a unique geographical location, as one part of GMS and southwestern gateway of China, moreover, surrounded by GMS high TB burden counties, making it indispensable to clarify the spatial-temporal heterogeneous distribution of TB in the province.

Our aim of this study was, first, to detect TB spatial-temporal clusters at county-level of Yunnan; second, to understand the temporal trend of notified TB in Yunnan; in addition, to explore correlated pattern of TB prevalence among GMS and border counties of Yunnan. Knowledge of long-term trend and spatial-temporal distribution of TB prevalence was crucial to understand the dynamic transmission of TB and to provide local evidence of classified TB prevention and control strategies.

## Methods

### General setting

**Fig. 1:**
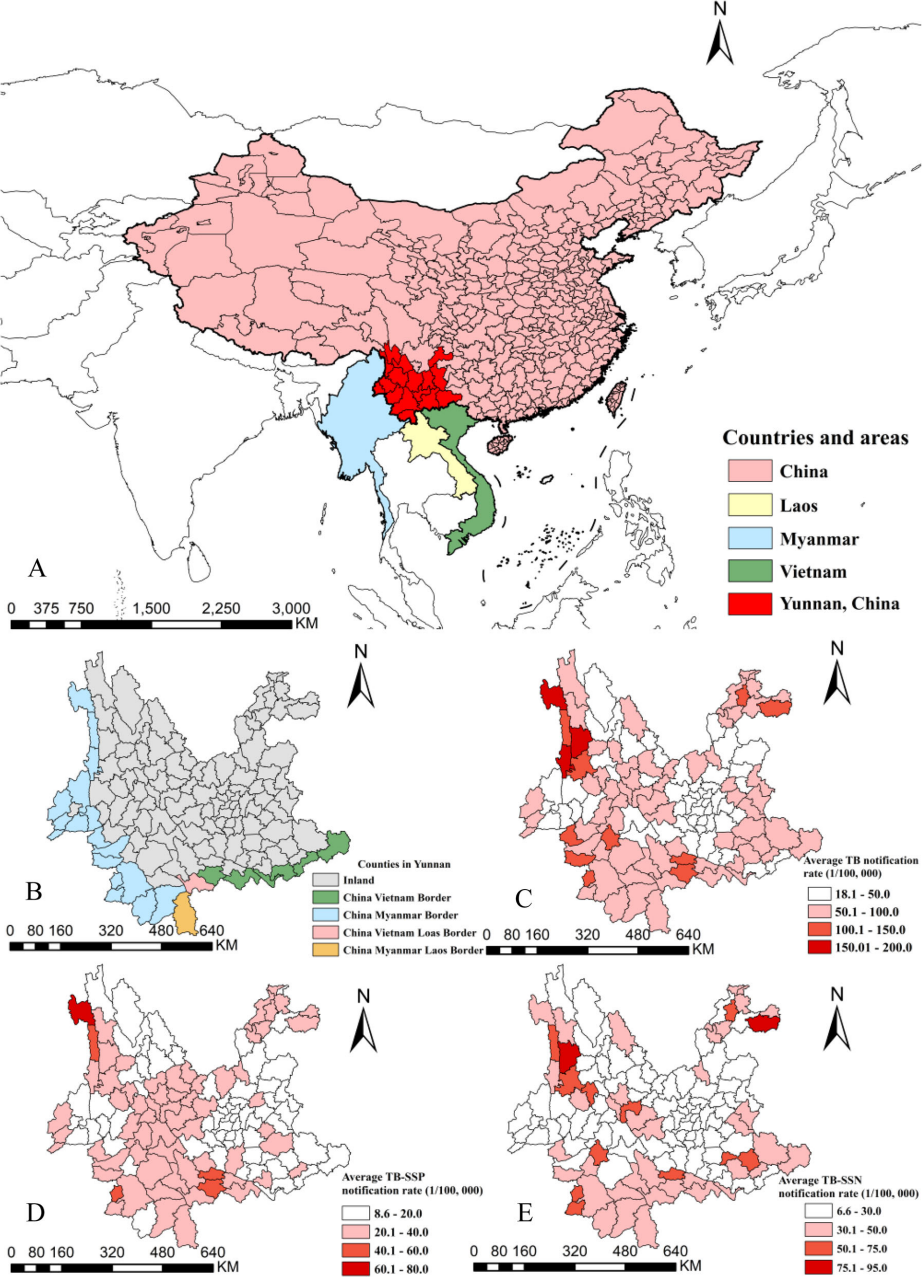
Location of Yunnan Province and Greater Mekong Subregion with border counties, and the county-level average tuberculosis prevalence of Yunnan, 2005–2018. Location and prefectures of Yunnan Province and China, Myanmar, Laos and Vietnam (**a**), the border and inland counties of China with GMS counties (**b**), The average prevalence of PTB in Yunnan, 2005–2018 (**c**), the average prevalence of SSP-TB in Yunnan, 2005–2018 (**d**), the average prevalence of SSN-TB in Yunnan, 2005–2018 (**e**). *GMS* Greater Mekong Subregion, *PTB* Pulmonary tuberculosis, *SSP-TB* Sputum smear-positive tuberculosis, *SSN-TB* Sputum smear-negative tuberculosis. World geographic database were extracted from the GADM database (https://www.gadm.org/), China and Yunnan geographic database provided by National Geomatics Center of China (http://www.ngcc.cn/ngcc/) at a 1:1,000,000 scale as the layer’s attribute

Yunnan locate in the far southwest corner of China, between longitude 97°31′ and 106°11′ East, latitude of 21°80′ and 29°15′ North, with a total area of 397,100 km^2^ while mountains occupy 84% of the territory, while the altitude various in tremendous range from 76 to 6740 m. There are 16 prefectures and 129 counties in Yunnan, the population of which was 48 million in 2018 [16]. Yunnan province compose GMS and adjoin Myanmar, Laos, and Vietnam by land (Fig. 1a). There are 25 border counties in Yunnan; 18, 2 and 7 counties of them adjoin with Myanmar, Laos, and Vietnam respectively. Mengla county adjoin by Myanmar and Laos, Jiangcheng county bordering on Laos and Vietnam simultaneously (Fig. 1b).

### Data source

TB diagnosis was based on patients’ suspicious symptoms plus results of Chest X-ray and sputum smear microscopy in TB designated hospital or county/district level Center for Disease Control and Prevention (CDC) TB clinic in Yunnan. Clinic diagnosed or laboratory confirmed PTB cases were notified in the China Information System for Disease Control and Prevention (CISDCP). Monthly TB cases notification data of 129 counties/districts in Yunnan from January 2005 to December 2018 were extracted from CISDCP. Annually demographic data of 129 counties/districts were collected from Yunnan statistical yearbooks from 2005 to 2018 [16]. The average TB notification rate was calculated by the numerator of summing county-level notified cases and the denominator of the summing population of counties in 14 years (Fig. 1 c, d, e). The raw counts and prevalence for PTB, SSP-TB, and SSN-TB (sputum smear-negative tuberculosis) cases were applied for analysis.

National-level estimated TB incidence and reported SSP-TB cases from 2005 to 2017 in GMS countries of Myanmar, Laos, and Vietnam were extracted from WHO’s global tuberculosis database (https://www.who.int/tb/data/en/).

### Statistical methods

#### Time series analysis

Monthly reported TB cases counts were aggregated into provincial level then analyzed by X-12-ARIMA (autoregressive integrated moving average) seasonal adjustment [5, 17], X-12-ARIMA was developed by US Census Bureau to define seasonal adjustment for time series [18]. By applying the X-12-ARIMA process, series of notified TB cases were decomposed into three components: seasonal variation, long-term trend cycle, and random irregular noise. Seasonal factors between 2005 to 2018 were calculated to explore seasonality of TB.

#### Temporal, spatial and space-time scan statistic

Kulldorff’s scan statistics was applied to detect clusters of TB cases in either a purely temporal, purely spatial or space-time setting. Spatial-temporal scan method created the infinite number of scanning windows, scan statistics gradually scanning a window across time and/or space, recorded the number of observed and expected observations inside the window at each location [19]. The scanning window was a time interval in the purely temporal scan for one-dimensional line, a circle in the purely spatial scan for bidimensional surface, or a cylinder in spatial-temporal scan with a circular base and height of time period for three-dimensional space [20, 21].

The surveillance data of notified TB case number against the population at risk in the same county/district was presumptively followed the Poisson probability distribution. Our study was a retrospective space-time scan analysis based on the discrete Poisson model. Under the Poisson distribution assumption, for each location and size of scanning windows, the alternative hypothesis was that there was an elevated risk within the window as compared to outside. The likelihood function calculated to define clusters, likelihood function was maximized overall scanned windows, maximum log-likelihood ratio (*LLR*) correspond the most likely cluster, which means the least likely to have occurred by chance; meanwhile, other ordinal statistically significant *LLRs* were matched to secondary clusters. *P*-value of maximum likelihood test was obtained through Monte Carlo hypothesis testing by randomly replicated simulations tests for comparing the rank of the maximum *LLR* from real data with from random data. The relative risk (*RR*) defined with the risk within scanned window compared to risk outside the scan window, *RR* representing how much more common disease was in this location and time period compared to the baseline [21].

Spatial-temporal scan parameters were selected by following the principle of reducing geographical overlap in clusters [10, 13]. The length of scanning time window covered 30% of the entire study period, and the scanning space window was set to cover 13% of the population at risk. Spatial scan for PTB prevalence in each year of study was applied to detect dynamic of geo-clusters, then scan spatial-temporal clusters for PTB, SSP-TB and SSN-TB prevalence by aggregating data of 14 years with the same parameters. The limit number of Monte Carlo replications was set to 999 times to detect the most likely and secondary clusters.

TB epidemic intensity in spatial-temporal clusters was measured by monthly average TB prevalence, which was calculated by the numerator of observed cases in cluster and denominator of population in the region multiply time interval (month) of the cluster.

#### Correlation and hierarchical clustering analysis

Chi-square tests were conducted to compare the proportion of PTB and SSP-TB purely spatial or spatial-temporal clusters between border and inland counties, then Pearson correlation coefficients were calculated by applying the PTB and SSP-TB prevalence of border counties or border counties within purely spatial clusters, Yunnan province, Myanmar, Laos, and Vietnam between 2005 to 2017. For the hierarchical clustering analysis, the first step was scaling and centering prevalence data; then define the similarity of prevalence by Euclidean distance; between-class distance and class agglomeration were defined by maximum between-class distance and complete linkage method; finally, the dendrogram presented the hierarchical clustering results.

The SaTScan™ software 9.6 (https://www.satscan.org) was applied for temporal, spatial, and spatial-temporal analysis. ArcGIS 10.2 (ESRI Inc) demonstrated geographical visualization for average TB prevalence and TB high-risk clusters. R software 3.5.2 (http://www.R-project.org) was applied for other statistical analysis. *P* < 0.05 indicates a statistical significance.

## Results

### Temporal distribution and clusters

**Fig. 2:**
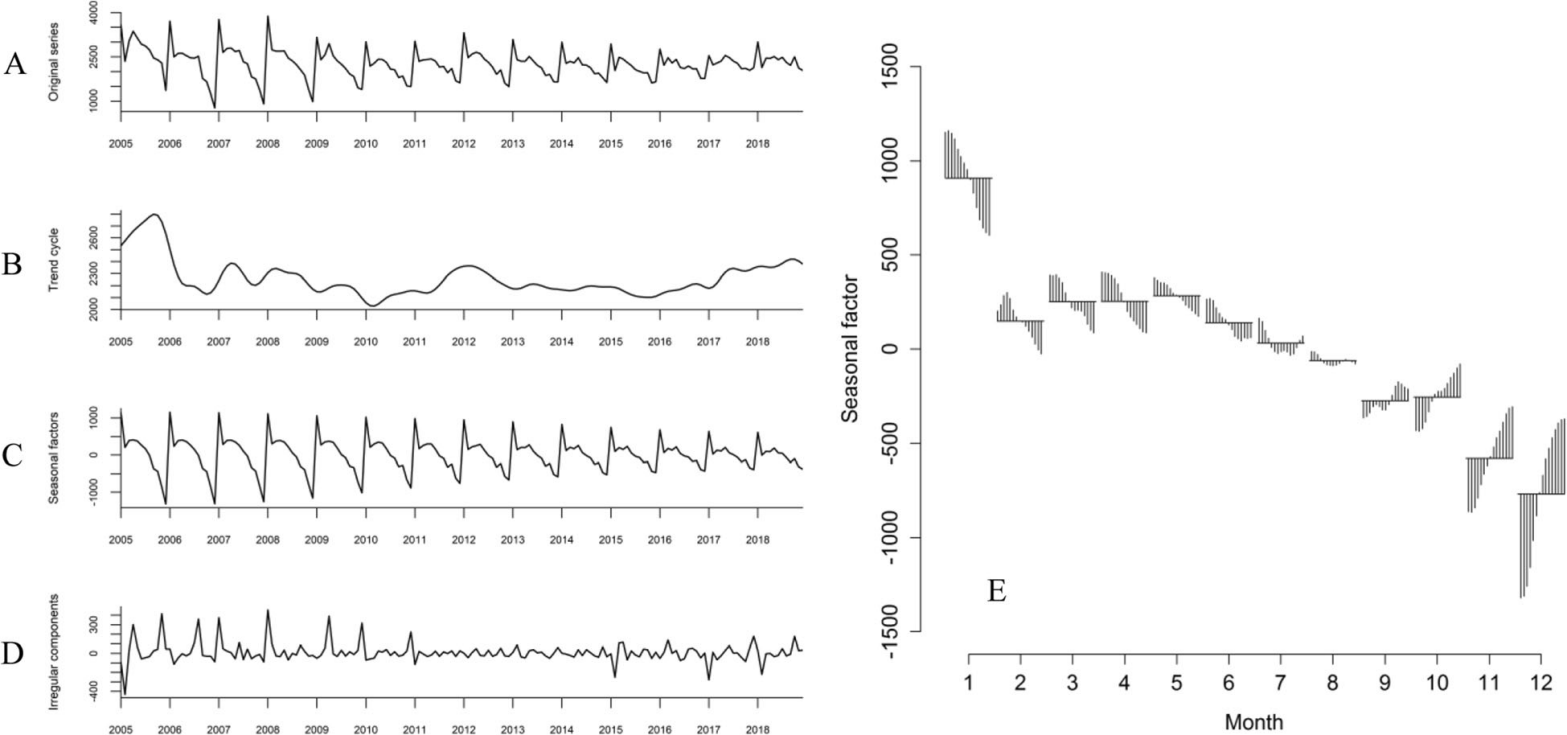
Seasonal time series decomposition of monthly tuberculosis cases in Yunnan, China, 2005–2018. Original series (**a**), Trend cycle (**b**), Seasonal factors (**c**), and irregular components (**d**). Irregular components: the residual that removed trend cycle and seasonal factor from original series

From 2005 to 2018, a total of 381,855 PTB cases were notified in Yunnan. The average PTB prevalence was 59.1 per 100,000 population, meanwhile, the average prevalence of SSP-TB and SSN-TB was 20.3 and 27.1 per 100,000 population respectively. Figure 2 showed the X-12-ARIMA seasonal decomposition of raw notified PTB counts, original series of PTB cases (Fig. 2a) with trend cycle (Fig. 2b), segregated seasonal factors (Fig. 2c), and irregular components (Fig. 2d). PTB trend cycle could be stated with 5 stages, beginning with a peak in 2005, a sharp downward trend between 2006 to 2011, followed by a rapid climb in 2011 to 2012, a steady fluctuation between 2013 to 2016, and a recent upward trend in 2017 to 2018. There was an obvious seasonality of notified PTB cases in isolated seasonal distribution, in each year the trough appeared in December, then sharply reached the peak in January. Though the seasonal factors declined in recent years, it periodically appeared across the entire study time frame of 14 years (Fig. 2e).

The annually temporal clusters included January except 2017 (Table 1), range of TB prevalence clusters concentrated in spring and/or summer. In the entire study time frame, temporal aggregation interval of PTB prevalence was in January 2005 to August 2006, for SSP-TB and SSN-TB the interval was from January 2008 to October 2011, and from January 2013 to February 2017 respectively (*P* < 0.01).

**Table 1:** Temporal clustering of pulmonary tuberculosis cases in Yunnan, 2005–2018

Categories	Year	Cluster time frame (start date to end date)	Observed cases (*n*)	Expected cases (*n*)	*RR*	*LLR*	*P*
PTB	2005	2005-1-1 to 2005-8-31	23,978	17,372	1.41	1181.73	< 0.001
PTB	2006	2006-1-1 to 2006-6-30	16,386	13,015	1.27	418.54	< 0.001
PTB	2007	2007-1-1 to 2007-6-30	17,331	13,114	1.34	639.52	< 0.001
PTB	2008	2008-1-1 to 2008-5-31	14,656	11,087	1.33	538.42	< 0.001
PTB	2009	2009-1-1 to 2009-4-30	11,055	8808	1.26	271.75	< 0.001
PTB	2010	2010-1-1 to 2010-1-31	2998	2288	1.31	100.96	< 0.001
PTB	2011	2011-1-1 to 2011-1-31	3022	2301	1.32	103.31	< 0.001
PTB	2012	2012-1-1 to 2012-5-31	13,594	11,372	1.20	210.77	< 0.001
PTB	2013	2013-1-1 to 2013-2-28	5487	4439	1.24	116.33	< 0.001
PTB	2014	2014-1-1 to 2014-1-31	2997	2346	1.28	83.53	< 0.001
PTB	2015	2015-1-1 to 2015-1-31	2938	2359	1.25	66.12	< 0.001
PTB	2016	2016-1-1 to 2016-1-31	2761	2374	1.16	30.21	< 0.001
PTB	2017	2017-5-1 to 2017-6-30	5020	4710	1.07	10.14	< 0.001
PTB	2018	2018-1-1 to 2018-1-31	3004	2403	1.25	70.12	< 0.001
PTB	2005–2018	2005-1-1 to 2006-8-31	53,814	43,601	1.27	1268.18	< 0.001
SSP-TB	2005–2018	2008-1-1 to 2011-10-31	45,941	35,513	1.45	1988.7	< 0.001
SSN-TB	2005–2018	2013-1-1 to 2017-2-28	69,538	53,061	1.52	3494.3	< 0.001

*PTB* Pulmonary tuberculosis, *SSP-TB* Sputum smear-positive tuberculosis, *SSN-TB* Sputum smear-negative tuberculosis, *RR* Relative risk, *LLR* Log-likelihood ratios

### Spatial distribution and clusters

**Fig. 3:**
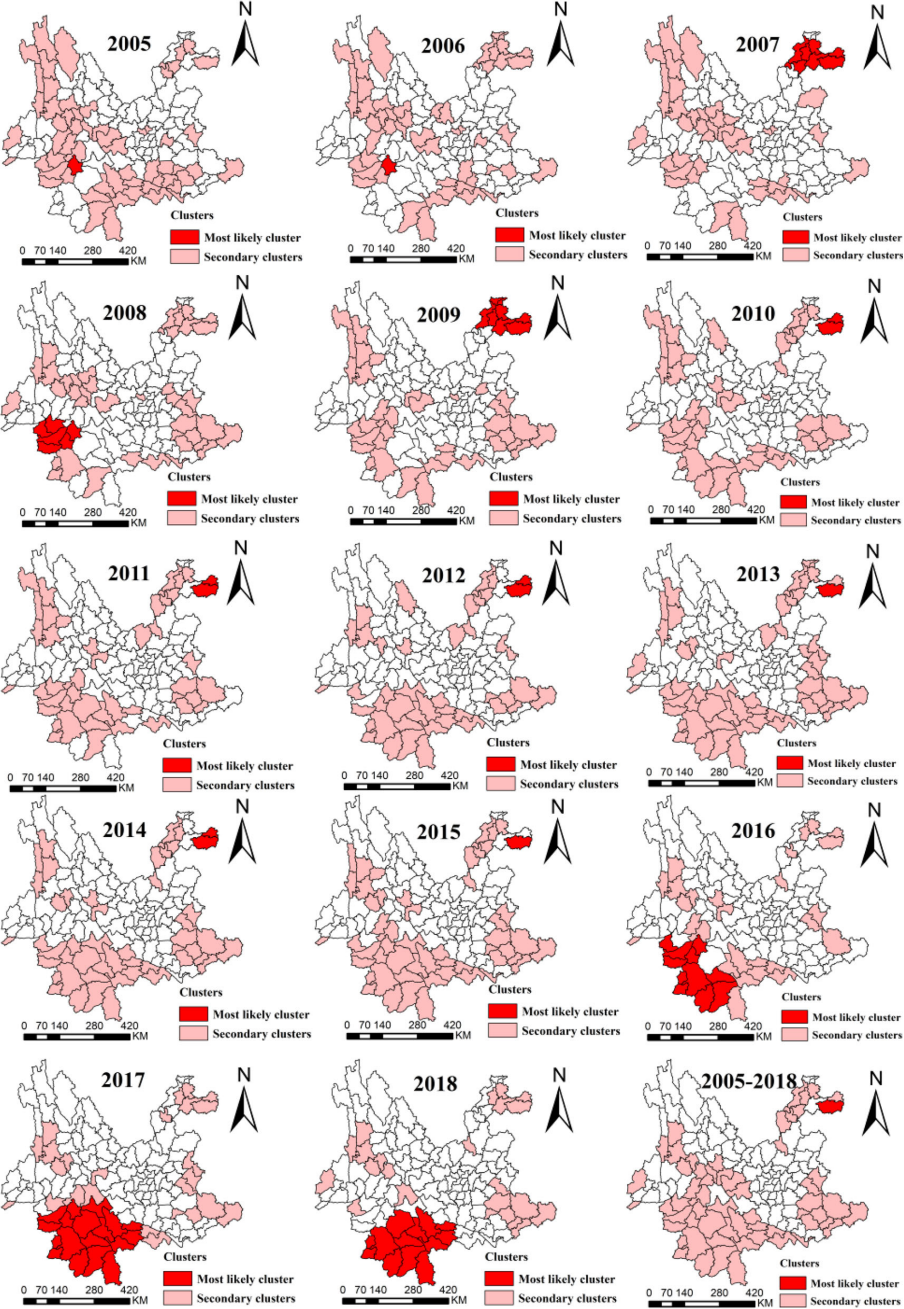
Spatial clustering of notified pulmonary tuberculosis cases in Yunnan, China, 2005–2018

The average prevalence of PTB, SSP-TB, and SSN-TB showed explicitly geographical heterogeneity in 129 counties of Yunnan from 2005 to 2018 (Fig. 1c,d,e). Annually spatial clustering analysis showed the dynamic process of PTB aggregations in space, the time-dependent most likely clusters were mainly concentrated in three regions and were shown in Fig. 3. In the year of 2005, 2006 and 2008, the cluster was in Linxiang county of Lincang prefecture. Another hotspot located in the northeast angle of Yunnan, which Zhenxiong and Weixin counties of Zhaotong prefecture were the center of clusters in 2007, and 2009 to 2015. Recently, the most likely cluster was in southwestern borders of Pu’er and Xishuangbanna prefectures between 2016 to 2018.

**Fig. 4:**
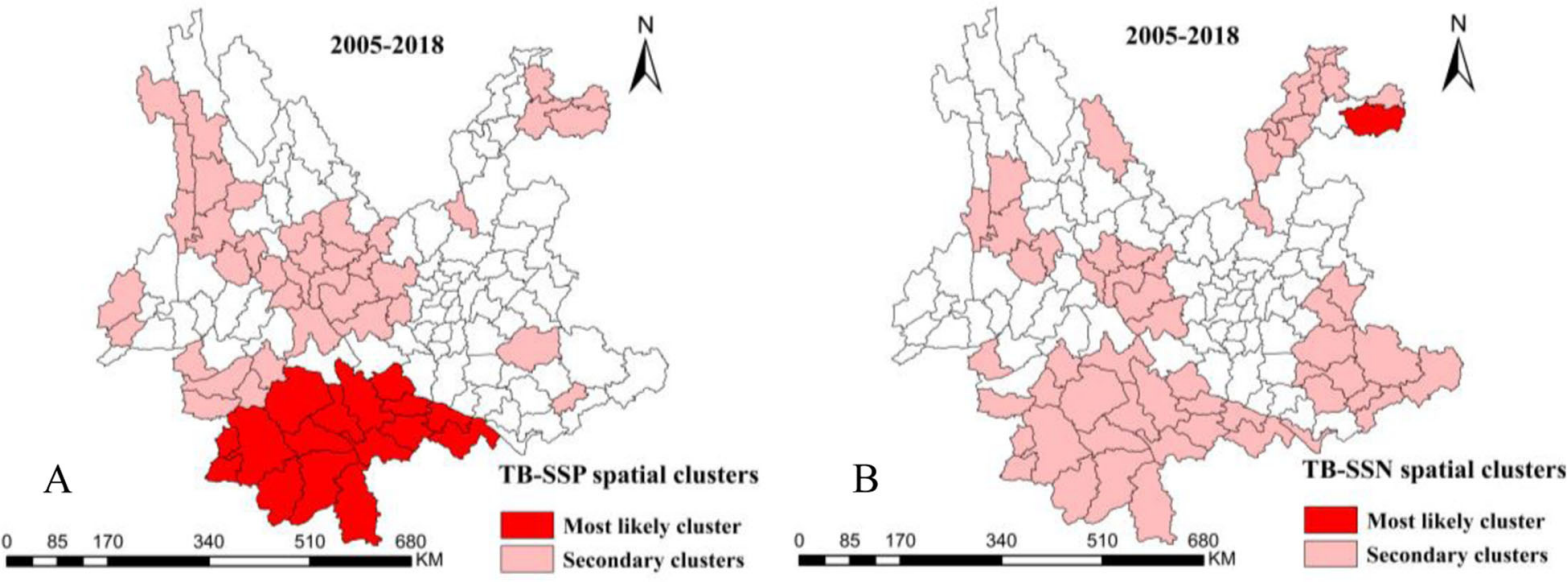
Spatial clustering of notified sputum smear-positive and smear-negative tuberculosis cases in Yunnan, China, 2005–2018. SSP-TB clusters (**a**) and SSN-TB clusters (**b**). *SSP-TB* Sputum smear-positive tuberculosis, *SSN-TB* Sputum smear-negative tuberculosis

The spatial clustering in the entire study period was similar to the annual aggregation, the most likely cluster of PTB and SSN-TB prevalence was Zhenxiong county between 2005 and 2018 (Fig. 3 and Fig. 4b), but the remarkable aggregation of SSP-TB was in the southwest frontier counties (Fig. 4a). Other secondary clusters scattered in the central, southeast and northeast of Yunnan.

### Spatial-temporal distribution and clusters

**Fig. 5:**
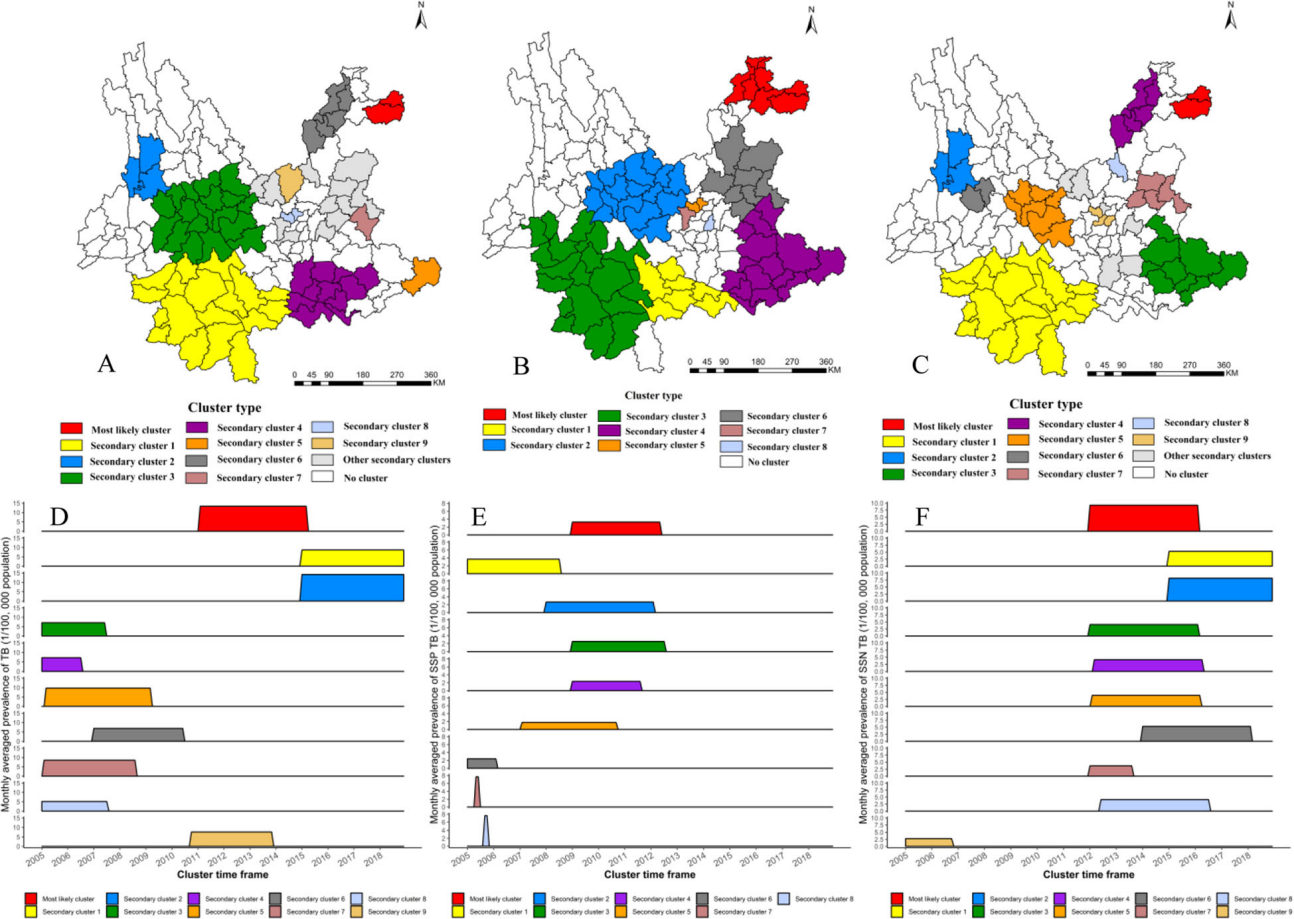
Spatial-temporal clustering of notified tuberculosis cases and monthly average tuberculosis prevalence of clusters in Yunnan, China, 2005–2018. Spatial-temporal clustering of notified PTB (**a**), Spatial-temporal clustering of notified SSP-TB (**b**), Spatial-temporal clustering of notified SSN-TB (**c**); Monthly average TB prevalence of PTB clusters (**d**), Monthly average TB prevalence of SSP-TB clusters (**e**), Monthly average TB prevalence of SSN-TB clusters (**f**). *TB* tuberculosis, *PTB* Pulmonary tuberculosis, *SSP-TB* Sputum smear-positive tuberculosis, *SSN-TB* Sputum smear-negative tuberculosis

The spatial-temporal distribution showed a consistent pattern with purely spatial scan for TB prevalence. Figure 5 and Table 2 showed the 17 spatial-temporal clusters for PTB, 9 clusters for SSP-TB, and 15 clusters for SSN-TB in 129 counties from 2005 to 2018. The most likely cluster for PTB was in northeast angle of Yunnan with coordinates of 27.90 N and 105.00 E, contained Zhenxiong and Weixin county in Zhaotong prefecture with a cluster circle radius of 43.1 Kilometers, which the high-risk period from February 2011 to March 2015 (*LLR* = 3657.6, *P* < 0.001). The risk was 2.6 times higher of developing active TB among residents within cluster compared with outside. A total of 10,963 PTB cases reported within the cluster and the average monthly prevalence was 13.4/100,000. Besides, there were the maximum reported cases (20080) and coverage counties (18 counties) in secondary cluster 1; the highest average monthly TB prevalence (14.2 per 100,000 population) in secondary cluster 2; specifically, both these two secondary clusters were in border region and were the latest clusters (Fig. 5a,d).

**Table 2:** Spatial-temporal clusters of pulmonary tuberculosis cases in Yunnan, 2005–2018

Cluster type	Cluster period	Coordinates/Radius	*N*	Observed cases (*n*)	Expected cases (*n*)	*RR*	*LLR*	*P*
Most likely cluster	2011-02-01 to 2015-03-31	(27.90 N, 105.00 E) / 43.10 km	2	10,963	4299	2.60	3657.56	< 0.001
Secondary cluster 1	2015-01-01 to 2018–12-31	(21.99 N, 100.31 E) / 240.95 km	18	20,080	11,739	1.75	2533.15	< 0.001
Secondary cluster 2	2015-01-01 to 2018–12-31	(26.03 N, 98.86 E) / 75.94 km	3	4097	1459	2.83	1600.85	< 0.001
Secondary cluster 3	2005-01-01 to 2007-06-30	(25.15 N, 100.55 E) / 119.09 km	18	12,399	8468	1.48	817.74	< 0.001
Secondary cluster 4	2005-01-01 to 2006-07-31	(23.30 N, 103.51 E) / 77.79 km	10	5034	3325	1.52	382.49	< 0.001
Secondary cluster 5	2005-03-01 to 2009-03-31	(23.66 N, 105.70 E) / 0 km	1	1953	992	1.97	362.91	< 0.001
Secondary cluster 6	2007-01-01 to 2010-06-30	(27.25 N, 103.41 E) / 87.96 km	5	6685	4731	1.42	362.18	< 0.001
Secondary cluster 7	2005-02-01 to 2008-08-31	(24.96 N, 104.32 E) / 0 km	1	2021	1160	1.75	261.64	< 0.001
Secondary cluster 8	2005-01-01 to 2007-07-31	(25.16 N, 102.66 E) / 14.67 km	2	2072	1247	1.67	228.04	< 0.001
Secondary cluster 9	2010–10-01 to 2013–11-30	(25.89 N, 102.58 E) / 0 km	1	1230	763	1.61	120.85	< 0.001
Secondary cluster 10	2006-01-01 to 2006-01-31	(25.76 N, 102.20 E) / 34.34 km	2	130	24	5.32	111.79	< 0.001
Secondary cluster 11	2009-08-01 to 2013-09-30	(26.15 N, 103.05 E) / 0 km	1	1140	718	1.59	104.95	< 0.001
Secondary cluster 12	2008-01-01 to 2008–10-31	(25.03 N, 103.71 E) / 0 km	1	590	307	1.92	102.22	< 0.001
Secondary cluster 13	2007-04-01 to 2007-07-31	(26.30 N, 104.13 E) / 0 km	1	467	273	1.71	56.96	< 0.001
Secondary cluster 14	2005-01-01 to 2006-09-30	(24.77 N, 103.42 E) / 0 km	1	407	241	1.69	47.25	< 0.001
Secondary cluster 15	2012-01-01 to 2012-05-31	(25.79 N, 103.86 E) / 62.11 km	4	709	514	1.38	33.06	< 0.001
Secondary cluster 16	2005-04-01 to 2005-06-30	(24.60 N, 102.53 E) / 27.58 km	2	142	85	1.67	15.81	0.012

*PTB* Pulmonary tuberculosis, *N* number of counties in the cluster, *RR* Relative risk,

*LLR* Log-likelihood ratios

Moreover, the spatial-temporal most likely cluster of SSP-TB and SSN-TB centered in northeastern Zhenxiong and Weixin county, particularly, the time frame of SSP-TB clusters aggregated before the year of 2012, though most of the SSN-TB clusters were detected after 2012 (Fig. 5b,c,e,f). More detail of these clusters could be found in additional files (Additional files 1 and 2).

### Correlation and hierarchical clustering analysis

**Fig. 6:**
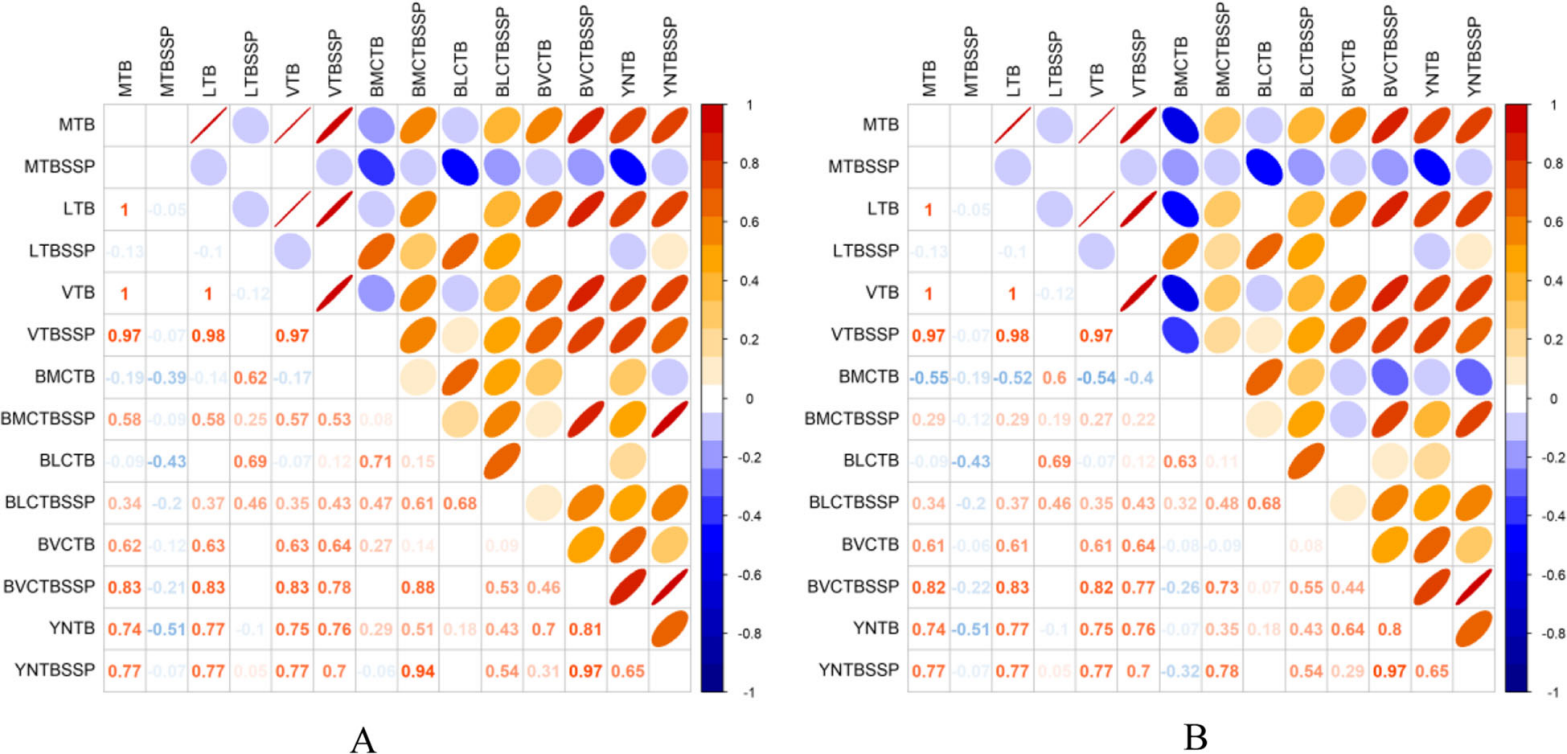
Correlation of tuberculosis prevalence among Greater Mekong Subregion and borders of Yunnan. All border counties of Yunnan (**a**), and border counties within spatial clusters (**b**). *GMS* Greater Mekong Subregion, *TB* Tuberculosis, *SSP-TB* Sputum smear-positive tuberculosis, *MTB* Myanmar TB incidence, *MTBSSP* Myanmar SSP-TB incidence, *LTB* Laos TB incidence, *LTBSSP* Laos SSP-TB incidence, *VTB* Vietnam TB incidence, *VTBSSP* Vietnam SSP-TB incidence, *BMCTB* Myanmar-China border TB prevalence, *BMCTBSSP* Myanmar-China border SSP-TB prevalence, *BLCTB* Laos-China border TB prevalence, *BLCTBSSP* Laos-China border SSP-TB prevalence, *BVCTB* Vietnam-China border TB prevalence, *BVCTBSSP* Vietnam-China border SSP-TB prevalence, *YNTB* Yunnan Province TB prevalence, *YNTBSSP* Yunnan Province SSP-TB prevalence

The association between regions and scanned clusters were shown in Table 3. For purely spatial scanning, a higher proportion of border counties were defined within PTB and SSP-TB clusters than inland counties(*P* < 0.01), meanwhile, for spatial-temporal scanning, there was no significant difference for the proportion of counties within and outside clusters between border and inland counties. Pairwise correlation coefficients between 25 border counties or 17 border counties within purely spatial clusters and GMS were shown in Fig. 6.

**Table 3:** Association between geographical regions and pulmonary tuberculosis clusters in Yunnan

Characteristic of clusters	Regions	Counties within clusters ^†^		Counties outside clusters ^‡^		*χ*^*2*^	*P*
		*n*	(*%*)	*n*	(*%*)		
Spatial clustering PTB cases							
	Border*	18	72.0	7	28.0	9.16	< 0.01
	Inland	40	38.5	64	61.5		
Spatial clustering SSP-TB cases							
	Border	17	68.0	8	32.0	8.71	< 0.01
	Inland	37	35.6	67	64.4		
Spatial clustering SSN-TB cases							
	Border	15	60.0	10	40.0	3.82	0.051
	Inland	40	38.5	64	61.5		
Spatial-temporal clustering PTB cases							
	Border	14	56.0	11	44.0	0.01	0.95
	Inland	59	56.7	45	43.3		
Spatial-temporal clustering SSP-TB cases							
	Border	16	64.0	9	36.0	0.30	0.56
	Inland	60	57.7	44	42.3		
Spatial-temporal clustering SSN-TB cases							
	Border	13	52.0	12	48.0	0.50	0.48
	Inland	46	44.2	58	55.8		

*PTB* Pulmonary tuberculosis, *SSP-TB* Sputum smear-positive tuberculosis, *SSN-TB* Sputum smear-negative tuberculosis,

† Counties within the most likely or secondary clusters

‡ Counties outside the most likely or secondary clusters

* Border: 25 border counties in Yunnan

**Fig. 7:**
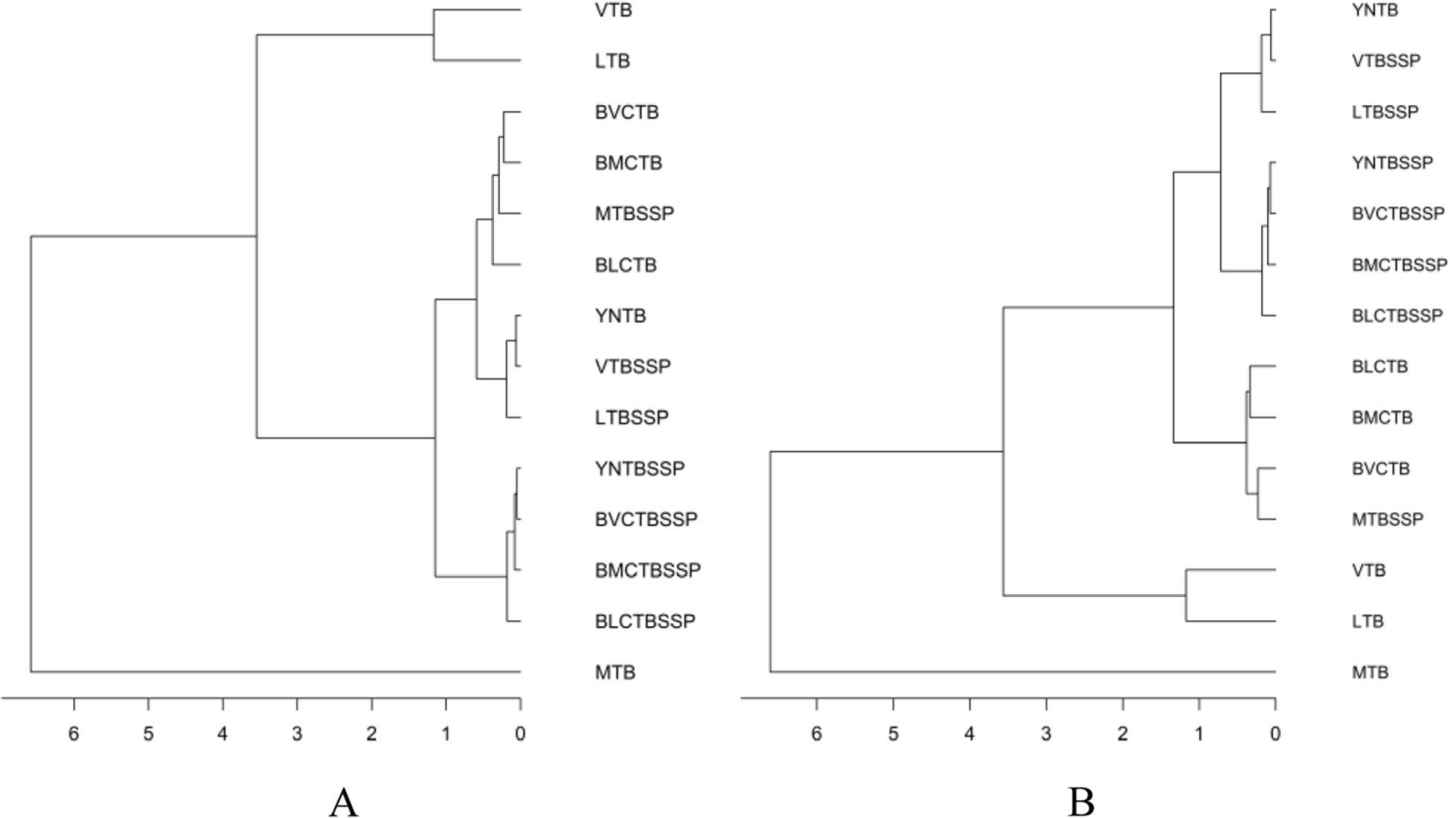
Hierarchical clustering dendrogram of tuberculosis prevalence pattern among Greater Mekong Subregion and borders of Yunnan. All border counties of Yunnan (**a**), and border counties within spatial clusters (**b**). *GMS* Greater Mekong Subregion, *TB* Tuberculosis, *SSP-TB* Sputum smear-positive tuberculosis, *MTB* Myanmar TB incidence, *MTBSSP* Myanmar SSP-TB incidence, *LTB* Laos TB incidence, *LTBSSP* Laos SSP-TB incidence, *VTB* Vietnam TB incidence, *VTBSSP* Vietnam SSP-TB incidence, *BMCTB* Myanmar-China border TB prevalence, *BMCTBSSP* Myanmar-China border SSP-TB prevalence, *BLCTB* Laos-China border TB prevalence, *BLCTBSSP* Laos-China border SSP-TB prevalence, *BVCTB* Vietnam-China border TB prevalence, *BVCTBSSP* Vietnam-China border SSP-TB prevalence, *YNTB* Yunnan Province TB prevalence, *YNTBSSP* Yunnan Province SSP-TB prevalence

Similarity patterns of TB prevalence defined by hierarchical clustering for 25 border counties or 17 border counties within purely spatial clusters with GMS were showed in Fig. 7. There were 6 possible patterns of TB prevalence among borders and GMS regardless of whether the borders were within purely spatial clusters. PTB incidence of Myanmar, Vietnam, Laos were independent categories; border counties PTB prevalence clustered with Myanmar SSP-TB prevalence; meanwhile, border counties SSP-TB prevalence were similar to Yunnan SSP-TB pattern; moreover, the pattern indicated that PTB prevalence of Yunnan highly correlated with Vietnam and Laos SSP-TB prevalence.

## Discussion

This study reveals temporal, spatial and spatial-temporal TB prevalence distribution in southwestern China at county-level in Yunnan province from 2005 to 2018. In brief, we detected TB high-risk time interval and high-epidemic areas for scanning spatial-temporal characteristics, yet identified similar patterns of TB prevalence among GMS. This study also presented the dynamic perspective of spatial-temporal PTB, SSP-TB and SSN-TB epidemic in Yunnan province at county-level.

The annual notification of PTB was 59.6 per 100,000 population of Yunnan in 2018. Although significant efforts made an annual decline rate of 1.5% from 2005, the high TB burden with absolute number of 28,618 cases reported in 2018, made a big challenge to achieve the goal of End TB in 2035 without the breakthrough of vaccine or new drug [22].

Time series decomposed secular trend showed that after PTB notification peak at 2005, the prevalence decreased first and then increased in recent years. Between 2003 and 2005, the detection of SSP-TB cases by the public health system more than doubled, from 30% of new cases to 80% [23]. The reason for the notification peak in 2005 was that China launched the direct internet-based reporting system for infectious diseases in 2004, thus greatly increased the TB reporting cases in 2005. After 2003, plenty efforts such as intensive DOTS implementation coverage, increased government commitment and improved public-health funding, all these measures focused on TB control lead to an acceleration of prevalence decline in the following decade [4, 24]. Recent years, active case finding strategy implementation increased the number of TB suspects with symptoms, which were 184,618 in 2018, almost doubled from 104,960 in 2015 [15, 25, 26], result in the current PTB prevalence upward in Yunnan between 2016 and 2018.

Seasonality was observed in different counties for TB notification. Interestingly, the peak month and trough month of TB notification were constant in the North Hemisphere regardless of the locations’ longitude. The peak months were roughly the same in American (spring, March) [27], South Korea (spring and summer) [28], Indian (spring, March to May) [29], Singapore (spring and summer, March and July) [30], China (spring, April) [5], Wuhan city (spring, March) [6] and Xingjiang autonomous prefecture of China (spring, March) [7]. Our study was consistent with these researches in the North Hemisphere, seasonal factors were observed and the peak in January and the secondary peak in May. The hypothesis of TB seasonality was related to the lack of sunshine and the lower temperature in winter. Vitamin D deficiency due to shorter daylight hours in winter [31], the temperature was inversely and lagged associated with TB incidence [32], all of which caused seasonality disease for the peak in spring and summer. In China, the Spring Festival effect should also be considered. Which means TB notification significant reduced during Spring Festival holidays, consequently, seasonal factors sharply declined in habitual Spring Festival month of February. Meanwhile, the purely temporal scan revealed the temporal clusters were concentrated in spring and summer in Yunnan each year. In the whole study time frame, the cluster interval for SSN-TB was from 2008 to 2011, though for SSN-TB was more recently from 2013 to 2017, which suggested the ongoing TB control policy should focus on SSN-TB in Yunnan.

Kulldorff’s scan statistics method was developed to evaluate temporal and geospatial distribution, it was applied to detect communicable disease, vector-borne diseases and cancer geospatial aggregation [33–36], meanwhile, the sensitivity of spatial-temporal statistics prompted early detection of disease outbreak and emergency disease from surveillance system [37, 38]. This powerful method showed the strength of statistical robustness and interpretability of analyzed results. Scan statistics were widely applied in study topic related to TB [39–43], whereas, data aggregated into large scales of administrative regions may ignore the disease variation in small size of population, information lose lead to inaccurate and insensitive conclusion [44], these national-level researches could not preciously detect localized cluster on the resolution of province or prefecture [9, 10, 45]. Meanwhile, due to the stochastic scan statistics sensitive to parameters, the analytical results on high-resolution scan of county-level may not stable. Small changes on the algorithm parameters lead to different results, especially in small size of population [13]. The fitness of setting the parameters is crucial to the analysis as a whole.

The purely spatial scan showed that the PTB in Yunnan were not randomly distributed, and the dynamic prevalence of PTB revealed three mainly aggregated regions, the hotspot of Yunnan north-eastern angle in Zhaotong prefecture was high frequently in clusters and hold 8 of 14 years in study interval. Previous studies examined the PTB clusters for Zhaotong prefecture were in towns of Zhengxiong and Weixin county [14, 46].

Spatial-temporal cluster pattern was in line with pure spatial scanning. Unexpectedly, spatial-temporal scan detected two clusters were implemented active cases finding, one was secondary cluster 2 (Lanping county) and another was cluster 11 (Dongchuan county) in PTB clusters. The time frame of clustering matched with activities of active cases finding [15, 47]. This suggested that by considering the cluster time interval, higher sensitivity and closer to reality outcome for the spatial-temporal scan. Besides, the time frame for SSP-TB clusters concentrated before 2012, though most of the SSN-TB clusters were defined after 2012, which indicated the decline of SSP-TB and the progress and achievement for tuberculosis control in Yunnan.

Our study found that the most recent cluster of PTB and the SSP-TB spatial cluster for the whole interval was in southwestern borders neighbored with Myanmar, Laos, and Vietnam. Furthermore, the correlation of TB prevalence among borders and GMS were relatively high. Strikingly, hierarchical clustering indicated that there were 6 subclasses for TB epidemic pattern among GMS, thus the borders’ TB prevalence was similar to Myanmar TB epidemic pattern. Based on the consistency of traditional and molecular epidemiology evidence which confirmed the relatively lower prevalence of Beijing genotype in the border region of Pu’er, Xishuangbanna, as well as Vietnam and Myanmar [48–50], we speculated that the residents living in the border region moved across the national boundary for livelihood while the air-borne disease of TB was carried beyond frontier. Recent high TB prevalence and high-risk temporal-spatial clusters in the GMS region suggested that cross-boundary intervention and international control policy should be implemented in these clusters.

Our study has some limitations. Firstly, the surveillance data did not contain covariates of patients’ demographic information for sex, age, etc., yet we did not introduce ecology factors like geographical, meteorological and economic situation, all of which could be possible indicators of TB incidence and prevalence. Secondly, we do not take account for unreported cases when using notifications data, since there was a risk of underestimated prevalence regardless of missing or unreported cases. Thirdly, it is difficult to collect the genetic and lower-level detailed geospatial information for TB among GMS, although it will advance the understanding of TB transmission among GMS. Further study should address these points.

## Conclusions

This study defined the long-term trend and seasonality of PTB prevalence for Yunnan province. Besides, we applied scan statistics to detect the temporal, spatial and spatial-temporal clusters of PTB prevalence between 2005 to 2018. The most likely cluster for PTB concentrated in the northeastern angle of Yunnan in distant past, the most recent spatial-temporal cluster of PTB and the spatial cluster of SSP-TB was in southwestern borders with GMS, meanwhile, the SSN-TB clusters were aggregated between 2012 to 2018. The similarity prevalence pattern of PTB among GMS suggested that priority of localized preventing implement should focus on cross-boundary intervention, yet the controlling strategy and resource allocation for the whole province should take account of the SSN-TB prevention.

## Supplementary information

**Additional file 1.** Spatial-temporal clusters of sputum smear-positive tuberculosis cases in Yunnan, 2005–2018.

**Additional file 2.** Spatial-temporal clusters of sputum smear-negative tuberculosis cases in Yunnan, 2005–2018.

## Data Availability

Data of the study was not publicly available, the datasets used and analysed during the current study are available from the corresponding author on reasonable request.

## Abbreviations

ARIMA: Autoregressive integrated moving average
CDC: Center for Disease Control and Prevention
China NTP: National Tuberculosis Control Program in China
CISDCP: China Information System for Disease Control and Prevention
DOTS: Directly observed treatment, short-course strategy
GMS: Greater Mekong Subregion
LLR: Log-likelihood ratio
RR: Relative risk
PTB: Pulmonary tuberculosis
SSN-TB: Sputum smear-negative tuberculosis
SSP-TB: Sputum smear-positive tuberculosis
TB: Tuberculosis
WHO: World Health Organization
