# Supplementary material for: The characteristics of spatial-temporal distribution and cluster of tuberculosis in Yunnan Province, China, 2005–2018
Source: BMC Public Health. 2019 Dec 21;19:1715. doi: 10.1186/s12889-019-7993-5 (PMC6925503; doi:10.1186/s12889-019-7993-5)
Supplement: Supplementary file 1 — Additional file 1. Spatial-temporal clusters of sputum smear-positive tuberculosis cases in Yunnan, 2005–2018. [file 12889_2019_7993_MOESM1_ESM.docx]

**Additional file 1.** Spatial-temporal clusters of sputum smear-positive tuberculosis cases in Yunnan, 2005-2018

| Cluster type |  | Cluster period |  | Coordinates/Radius |  | *N* |  | Observed cases  (*n*) | | Expected cases  (*n*) | |  | *RR* |  | *LLR* | *P* |
| --- | --- | --- | --- | --- | --- | --- | --- | --- | --- | --- | --- | --- | --- | --- | --- | --- |
| Most likely cluster |  | 2009-1-1 to 2012-5-31 |  | (28.512421 N, 104.245850 E) / 119.22 km |  | 8 |  | 4541 |  | 2355 |  |  | 1.96 |  | 814.10 | <0.001 |
| Secondary cluster 1 |  | 2005-1-1 to 2008-7-31 |  | (22.847942 N, 102.218325 E) / 91.75 km |  | 7 |  | 3096 |  | 1400 |  |  | 2.24 |  | 771.62 | <0.001 |
| Secondary cluster 2 |  | 2008-1-1 to 2012-2-29 |  | (25.520084 N, 101.253635 E) / 112.36 km |  | 16 |  | 6557 |  | 4202 |  |  | 1.59 |  | 584.82 | <0.001 |
| Secondary cluster 3 |  | 2009-1-1 to 2012-7-31 |  | (22.697727 N, 99.513073 E) / 218.58 km |  | 19 |  | 6034 |  | 4157 |  |  | 1.47 |  | 385.36 | <0.001 |
| Secondary cluster 4 |  | 2009-1-1 to 2011-8-31 |  | (23.666653 N, 105.708450 E) / 227.34 km |  | 14 |  | 4125 |  | 3001 |  |  | 1.39 |  | 193.19 | <0.001 |
| Secondary cluster 5 |  | 2007-2-1 to 2010-9-30 |  | (25.165231 N, 102.665035 E) / 14.67 km |  | 2 |  | 1017 |  | 624 |  |  | 1.63 |  | 104.40 | <0.001 |
| Secondary cluster 6 |  | 2005-1-1 to 2006-2-28 |  | (25.794895 N, 103.869082 E) / 85.59 km |  | 8 |  | 1796 |  | 1258 |  |  | 1.43 |  | 102.50 | <0.001 |
| Secondary cluster 7 |  | 2005-5-1 to 2005-6-30 |  | (24.816926 N, 102.395590 E) / 0 km |  | 1 |  | 49 |  | 10 |  |  | 4.80 |  | 38.03 | <0.001 |
| Secondary cluster 8 |  | 2005-9-1 to 2005-10-31 |  | (24.709781 N, 102.925780 E) / 0 km |  | 1 |  | 25 |  | 5 |  |  | 4.74 |  | 19.18 | <0.001 |

SSP-TB Sputum smear-positive tuberculosis, *N* number of counties in the cluster,

*RR* Relative risk, *LLR* Log-likelihood ratios
