# Supplementary material for: The characteristics of spatial-temporal distribution and cluster of tuberculosis in Yunnan Province, China, 2005–2018
Source: BMC Public Health. 2019 Dec 21;19:1715. doi: 10.1186/s12889-019-7993-5 (PMC6925503; doi:10.1186/s12889-019-7993-5)
Supplement: Supplementary file 2 — Additional file 2. Spatial-temporal clusters of sputum smear-negative tuberculosis cases in Yunnan, 2005–2018. [file 12889_2019_7993_MOESM2_ESM.docx]

**Additional file 2.** Spatial-temporal clusters of sputum smear-negative tuberculosis cases in Yunnan, 2005-2018

| Cluster type |  | Cluster period |  | Coordinates/Radius |  | *N* |  | Observed cases  (*n*) | | Expected cases  (*n*) | |  | *RR* |  | *LLR* | *P* |
| --- | --- | --- | --- | --- | --- | --- | --- | --- | --- | --- | --- | --- | --- | --- | --- | --- |
| Most likely cluster |  | 2012-1-1 to 2016-2-29 |  | (27.904794 N, 105.003335 E) / 43.10 km |  | 2 |  | 7614 |  | 1994 |  |  | 3.95 |  | 4674.01 | <0.001 |
| Secondary cluster 1 |  | 2015-1-1 to 2018-12-31 |  | (21.990198 N, 100.318620 E) / 240.95 km |  | 18 |  | 12228 |  | 5382 |  |  | 2.37 |  | 3329.00 | <0.001 |
| Secondary cluster 2 |  | 2015-1-1 to 2018-12-31 |  | (26.038454 N, 98.860439 E) / 75.94 km |  | 3 |  | 2352 |  | 669 |  |  | 3.55 |  | 1282.07 | <0.001 |
| Secondary cluster 3 |  | 2012-1-1 to 2016-2-29 |  | (23.980826 N, 105.054250 E) / 132.08 km |  | 9 |  | 8307 |  | 4702 |  |  | 1.80 |  | 1160.98 | <0.001 |
| Secondary cluster 4 |  | 2012-3-1 to 2016-4-30 |  | (27.257782 N, 103.411205 E) / 87.96 km |  | 5 |  | 4789 |  | 2699 |  |  | 1.80 |  | 668.27 | <0.001 |
| Secondary cluster 5 |  | 2012-2-1 to 2016-3-31 |  | (25.038232 N, 101.227780 E) / 73.33 km |  | 7 |  | 4211 |  | 2505 |  |  | 1.70 |  | 489.62 | <0.001 |
| Secondary cluster 6 |  | 2014-1-1 to 2018-2-28 |  | (25.399637 N, 99.610562 E) / 31.32 km |  | 2 |  | 766 |  | 325 |  |  | 2.36 |  | 215.95 | <0.001 |
| Secondary cluster 7 |  | 2012-1-1 to 2013-8-31 |  | (25.794895 N, 103.869082 E) / 62.11 km |  | 4 |  | 1516 |  | 947 |  |  | 1.61 |  | 145.19 | <0.001 |
| Secondary cluster 8 |  | 2012-6-1 to 2016-7-31 |  | (26.154414 N, 103.051690 E) / 0 km |  | 1 |  | 603 |  | 312 |  |  | 1.94 |  | 106.71 | <0.001 |
| Secondary cluster 9 |  | 2005-1-1 to 2006-10-31 |  | (24.950452 N, 102.640870 E) / 23.99 km |  | 3 |  | 990 |  | 616 |  |  | 1.61 |  | 96.21 | <0.001 |
| Secondary cluster 10 |  | 2006-1-1 to 2006-1-31 |  | (25.761661 N, 102.200565 E) / 34.34 km |  | 2 |  | 56 |  | 11 |  |  | 5.00 |  | 45.34 | <0.001 |
| Secondary cluster 11 |  | 2017-3-1 to 2018-8-31 |  | (23.743769 N, 103.387465 E) / 0 km |  | 1 |  | 258 |  | 137 |  |  | 1.89 |  | 42.77 | <0.001 |
| Secondary cluster 12 |  | 2006-1-1 to 2006-8-31 |  | (23.705351 N, 102.889395 E) / 0 km |  | 1 |  | 195 |  | 97 |  |  | 2.00 |  | 37.78 | <0.001 |
| Secondary cluster 13 |  | 2005-3-1 to 2006-9-30 |  | (25.161341 N, 102.810789 E) / 0 km |  | 1 |  | 249 |  | 164 |  |  | 1.52 |  | 18.78 | <0.001 |
| Secondary cluster 14 |  | 2005-4-1 to 2006-4-30 |  | (24.773820 N, 103.423190 E) / 0 km |  | 1 |  | 123 |  | 68 |  |  | 1.80 |  | 17.60 | <0.001 |

SSN-TB Sputum smear-negative tuberculosis, *N* number of counties in the cluster,

*RR* Relative risk, *LLR* Log-likelihood ratios
